# Altered physiochemical properties in industrially synthesized ZnO nanoparticles regulate oxidative stress; induce *in vivo* cytotoxicity in embryonic zebrafish by apoptosis

**DOI:** 10.1038/s41598-017-14039-y

**Published:** 2017-10-24

**Authors:** Suresh K. Verma, Pritam Kumar Panda, Ealisha Jha, Mrutyunjay Suar, S. K. S. Parashar

**Affiliations:** 1 School of Biotechnology, KIIT University, 751024 Bhubaneswar, India; 2 School of Applied Sciences, KIIT University, 751024 Bhubaneswar, India

## Abstract

This study investigates the *in vivo* cytotoxicity of ZnO nanoparticles synthesized at industrial scale with embryonic Zebrafish. Industrial synthesis of ZnO nanoparticles was mimicked at lab scale by high energy ball milling technique by milling bulk ZnO particles for 15 h. Synthesized 7 h and 10 h ZnO nanoparticles showed significant alteration of size, zeta potential and optical properties in comparison to Bulk ZnO. Mortality and hatching rate in Zebrafish embryos were influenced by these alterations. Size and charge dependent effect of ZnO nanoparticles exposure on physiology and development of Zebrafish embryos were evident by malfunctioned organ development and abnormal heartbeat rate. Similar dependency on quenching of ROS due to influential hydrogen bond interaction with glycine residue of Sod1 oxidative stress protein and increased apoptosis were observed in cells. The study revealed the mechanism of cytotoxicity in exposed embryonic Zebrafish as an effect of accumulation and internalization inside cells instigating to generation of hypoxic condition and interference with the normal adaptive stress regulation signaling pathways leading towards enhanced apoptosis. The study revealed hidden size and charge dependent *in vivo* cytotoxicity mechanism of ZnO nanoparticles in Zebrafish embryos insight of the environmental and clinical importance of attention on industrially synthesized ZnO nanoparticles.

## Introduction

ZnO nanoparticles have been recognized as one of the most attentive nanoparticles in last few decades of nanotechnology science. It has got this reputation because of its peculiar physiochemical properties^1–4^ and wide aspects of applications including production of wave fitters, UV detectors, catalysis, paint, transparent conductive field, cosmetics, gas sensors, antibacterial agent and microelectronics^5–10^. It has also been used as important constituents in products like personal care products (toothpaste, beauty products and sunscreen)^11,12^ and textiles products^13,14^. Moreover, it has been reported as a potent applicant in medicine for infectious^15–18^ and non-infectious diseases^19^. With this extensive usage of these nanoparticles, much efforts and research have been done by researchers to synthesize ZnO nanoparticles with application based properties^16,20–22^. It has been established that different forms of nano ZnO can be synthesized by controlling the different synthesis parameters^23^. Many researchers have discovered and established different methods and route for synthesis of different types of ZnO nanoparticles. Some of the reported methods includes chemical methods^24,25^, physical methods^26–29^ and biological techniques^30–32^. Efforts by chemical route has been made on synthesis of ZnO nanoparticles with desired physiochemical properties^33^ while biological synthesis has been implicated for formation of biocompatible nanoparticles^34^. For large scale synthesis in industrial application physical route has been preferred. Physical route of nano ZnO synthesis includes use of physical factors like heat^35–37^, mechanical force etc. Among these various techniques mechanical route of synthesis is most popular and commonly called as “High energy ball milling (HEBM)” technique^38,39^. HEBM has been reported as an important technique for synthesizing ZnO nanoparticles in industrial scale without involving any complex chemical synthesis^38^. Advantage of HEBM over other reported methods has been proved in terms of simplicity, reliability and reproducibility^40,41^. It has been proved to induce rare chemical reaction at room temperature^41^. Moreover it has been reported to induce changes in electrical and optical properties along with morphological and structural alterations. Due to this high reliability and perfectness, HEBM technique is used in industries for large scale production of nanoparticles.

With increase in extensive usage of ZnO nanoparticles, concerned have risen for their eco-toxicological and toxicological effects. Studies have been done to investigate the *in vitro* cytotoxic effect of ZnO and other metallic nanoparticles in different mammalian cell line system to discover and illustrate the mechanism of cytotoxicity^42–46^. It has been reported that ZnO and other metallic nanoparticles exhibit cytotoxicity^47^ and genotoxicity^48^ in different cells by enhancing ROS production^49–51^ and alternating the different metabolism leading to DNA damage^52–54^, malfunctioning of cell organelles^55,56^ and ultimately their death. *In vivo* cytotoxicity has also been investigated through different animal models like Zebrafish and Mouse^57–59^. It has been stated that cytotoxicity of Zinc oxide nanoparticles is dependent on their shape, size and other physiochemical properties and it affects the morphological development and cellular metabolism in Zebrafish and mice^59,60^. These investigation reports are based upon the testing of ZnO nanoparticles prepared at lab scale through different methods. However, information about the cytotoxicity and genotoxicity of ZnO nanoparticles synthesized at industrial scale is still not mentioned anywhere. With this study, we have tried to fill that information gap by investigating those effects for the first time. ZnO nanoparticles were prepared by HEBM technique as prototype of industrial scale synthesis and their *in vivo* cytotoxicity was investigated for the first time to show their impact in real case.

Zebrafish (*Danio rerio*) has been considered as one of the most potential model organism for toxicity of assessment of nanoparticles in last few years. Developing Zebrafish embryos has been proved to be perfect live model for investigation of effect of any pollutant or nanoparticles because of its transparency and fast life cycle^61,62^. Moreover it is easy to maintain at laboratory with cost effectiveness as compared to other models. Toxicity reports for any nanoparticles exposed to embryo zebrafish can be considered to mimic their effect on human because of their genetic and physiological similarities with human^63,64^. Many literatures have reported *in vivo* cytotoxicity assessment of different engineered nanoparticles through Zebrafish model^65–67^. Moreover, toxicity assessment in Zebrafish model can be beneficial in duel way by considering the reports for ecological assessment as well as for human health.

The goal of this study is to explore the physiochemical changes in industrially prepared ZnO nanoparticles and investigate consequent effect of their exposure on living cells especially to aquatic animals. ZnO nanoparticles were synthesized in lab scale by HEBM technique considering it as a prototype of industrial method. *In vivo* cytotoxicity of synthesized ZnO nanoparticles was determined at cellular and physiological level with the help of Zebrafish model and the probable mechanism was discussed insight of experimental and computational observation and previous reports.

## Materials and Methods

### Synthesis of ZnO nanoparticles

Synthesis of Zinc Oxide (ZnO) nanoparticles were done by milling Bulk Zinc Oxide powder (Merck) in planetary High Energy ball Milling (Retsch, PM400) using tungsten carbide (WC) jar (250 ml) and WC ball (10 mm) at 300 rpm with ball to powder ratio 20:1 in ambient atmosphere from 0 to 10 h. For cytotoxicity analysis, suspension of Bulk ZnO, 7 h and 10 h milled ZnO nanoparticles was prepared in Holtfreter medium (HF)^68^ by sonication at amplitude of 50 for 5 minutes with no pulse.

### Characterization of synthesized ZnO nanoparticles

Characterization for physiochemical properties of synthesized ZnO nanoparticles were performed by standard techniques. Size and shape of the ZnO nanoparticles were determined by visualization with Field emission scanning electron microscope (FESEM). For imaging, ZnO nanoparticles were suspended in HF medium by sonication and air dried on a silicon substrate. Imaging was performed using a Carl Zeiss, Neon 40 microscope at 20KV equipped with EDS (Inca, Oxford). Structural analysis was performed by XRD techniques using an X-ray diffractometer (X-PERT- PRO, Pan Analytical) with CuKα radiation (λ = 0.15418 nm) over wide range of angle of 15° to 75°. The average size of the milled ZnO nanoparticles was calculated from X-ray peak broadening using Voigt peak profile analysis, after eliminating the instrumental broadening and strain contribution. Size and stability of ZnO nanoparticles in HF medium was tested by measuring their hydrodynamic diameter and zeta potential with the help of Dynamic light scattering (Zetasizer nanoseries, Malvern, UK). Optical properties of ZnO nanoparticles were determined by UV-Vis spectroscopy measuring optical density at a range of 300–800 nm.

### Zebrafish maintenance and embryo culture

All animal procedures were approved by the relevant guidelines of Institutional Animal Ethics Committee (IAEC) of KIIT University. All experiments were performed in accordance with relevant animal practice guidelines and regulations of IAEC, KIIT University. Maintenance of adult Zebrafish (Danio rerio) was done in overflow container setup supplied by Aquaneering, USA. The setup was equilibrated with fish water (75 g NaHCO_3_, 18 g sea salt, 8.4 g CaSO_4_ per 1000 ml). Feeding of fishes was done thrice a day with fish food containing bloodworm. Embryos were obtained by breeding male and female placed in setup box containing net partition in a ratio of 2:1. 12 h light and 12 h dark were maintained for photoperiods. The eggs were spawned after 2 hour after removing the partition. Viable eggs were collected and rinsed with Holtfreter (HF) medium. Embryos were maintained in HF medium for further experiments. All the chemicals used for preparing buffer were purchased from Sigma Aldrich.

### Embryo and larvae cytotoxicity assays

All protocols were approved by relevant guidelines of IAEC of KIIT University. Embryos of 3–3.5 hour post fertilization (hpf) at blastula stage were selected for different toxicity assays. Toxicity assays conducted with Zebrafish embryo exposed to synthesized ZnO nanoparticles were performed by protocol mentioned by Scholz *et al*.^69^. Zebrafish embryos were treated with different concentration of Bulk and ZnO nanoparticles for 96 h in 24 wells plate containing HF buffer. The experimental set up was exposed to photoperiod of 14/10 h light/dark at 28 ± 1 °C. Untreated embryos were taken as control. Morphological changes were observed by direct observation with stereomicroscope. Hatching rate was determined as number of embryos hatched by 96 h post fertilization as compared to untreated group. Survivability rate was expressed as number of live embryos after 96 h post fertilization as compared to untreated group. Heart rate was calculated for each group by counting. All the experiments were performed in triplicates and repeated thrice.

### Examination of cellular reactive oxygen species (ROS)

To determine the mechanism underlying synthesized ZnO nanoparticle toxicity intracellular ROS was investigated. Intracellular ROS was measured by flow cytometry and Fluorescent microscopy using 2,7-dichlorodihydrofluorescein diacetate (H_2_DCFDA) fluorescent dye (Sigma, Aldrich) which is a permanent ROS marker^70^. Untreated and 50 µg/ml of Bulk, 7 h and 10 h ZnO nanoparticles treated 96 h hatched embryos were washed with HF buffer and stained with H_2_DCFDA for 20 min in dark. The fluorescence was observed with microscope at green channel and images were taken. For flow cytometry analysis, live hatched embryos were sacrificed and single suspension of cells was prepared by sonication. Then, cell suspension was stained with 1.25 mg/l H_2_DCFDA dye for 20 min in dark followed by washing with 1X phosphate buffer solution. The cells were subsequently analyzed by flow cytometry using Attune acoustic focusing cytometer (Applied Biosystems, Life technologies) equipped with 488 nm argon laser. The data were analyzed in facsxpress 5 (Denovo, CA) and presented as histogram. All the experiments were performed in triplicates and significance was calculated using Graph pad prism6.

### *In silico* analysis of ZnO-oxidative stress protein interaction

In order to determine the interaction of oxidative stress proteins with ZnO nanoparticles, *in silico* approach was taken. Molecular docking was performed using ZnO as ligand and sod1 protein molecule as receptor protein. Molecular docking studies were carried out using Auto Dock Vina 4.2^71^ Blind Docking was performed with the Grid Box cube of 40 and spacing of 1 with the x, y, and z centers as −24.301, 49.934, 3.896. Preparation of protein was carried out using these parameters and by adding Kollman charges^72^ and merging non-polar hydrogen atoms. The ligands were prepared using Autodock by applying Gasteiger charges and merging non-polar hydrogen atoms. 9 poses were generated for each ligand based on the best-suited orientation of the molecule. The molecular docking scores of sod1 with ZnO were tabulated. The hydrogen-bonding patterns were visualized using Chimera and Ligplot^+^^73^.

### Acridine orange staining for apoptosis analysis

Analysis of Apoptosis in 96 h zebrafish embryo exposed to 50 µg/ml Bulk,7 h and 10 h ZnO nanoparticles was performed with the help of Acridine orange staining protocol mentioned by Asharani *et al*.^74^. In brief, untreated and treated zebrafish embryos were washed two times with HF buffer after treatment and stained with 5 µg/ml AO dissolved in HF for 20 min. Followed by staining, embryos were washed with sterilized HF buffer to remove extra stain and observed in green channel of fluorescent microscope (EVOS, ThermoScientific).

## Results

### Characterization of Synthesized ZnO nanoparticles

**Figure 1:**
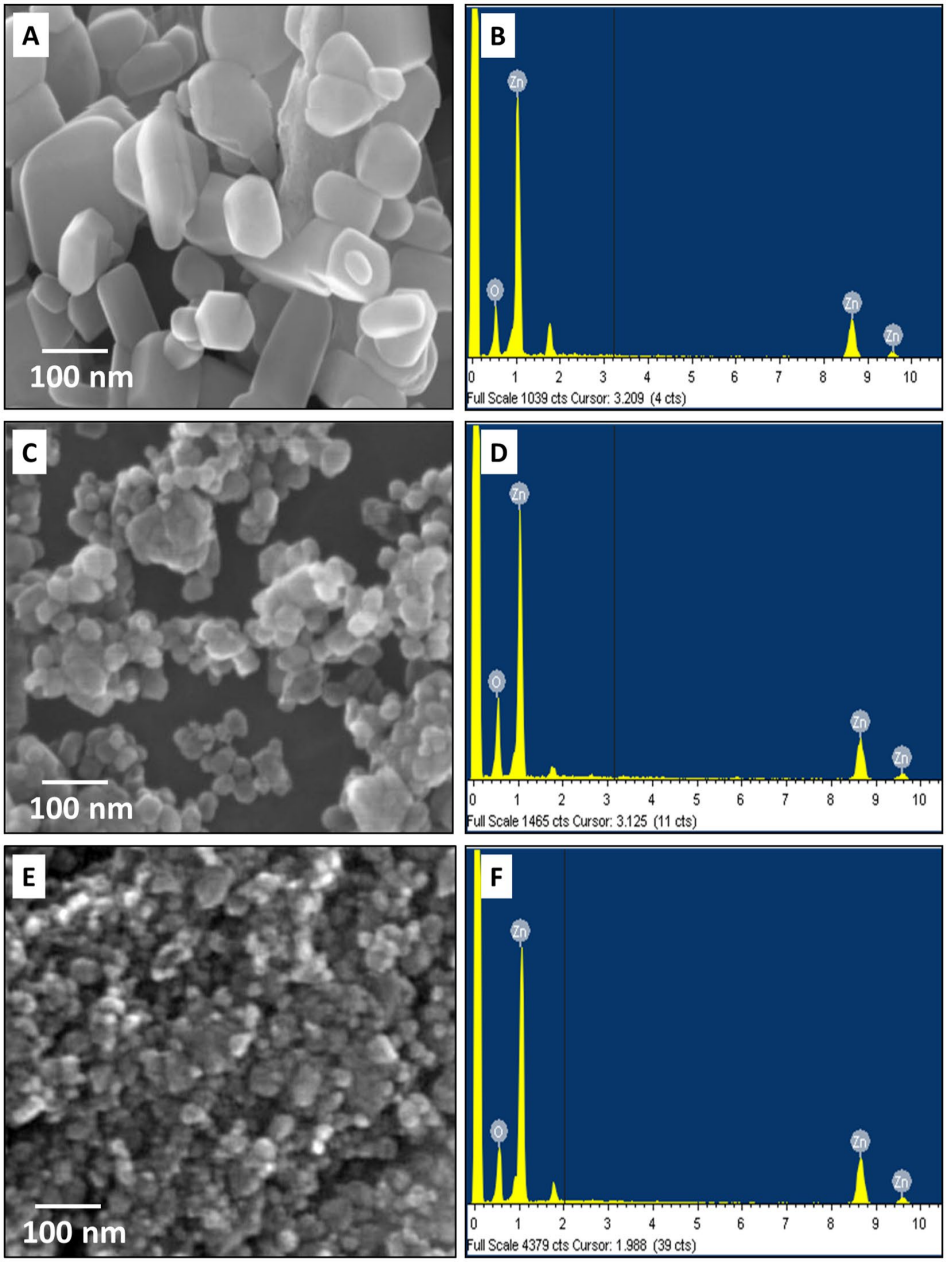
FESEM image of HEBM synthesized ZnO nanoparticles milled at different time. (**A**) Bulk (0 h) ZnO (**C**) 7 h nano ZnO (**E**) 10 h nano ZnO. (**B**) Bulk (**D**) 7 h (**F**) 10 h shows EDS analysis of ZnO nanoparticles.

**Figure 2:**
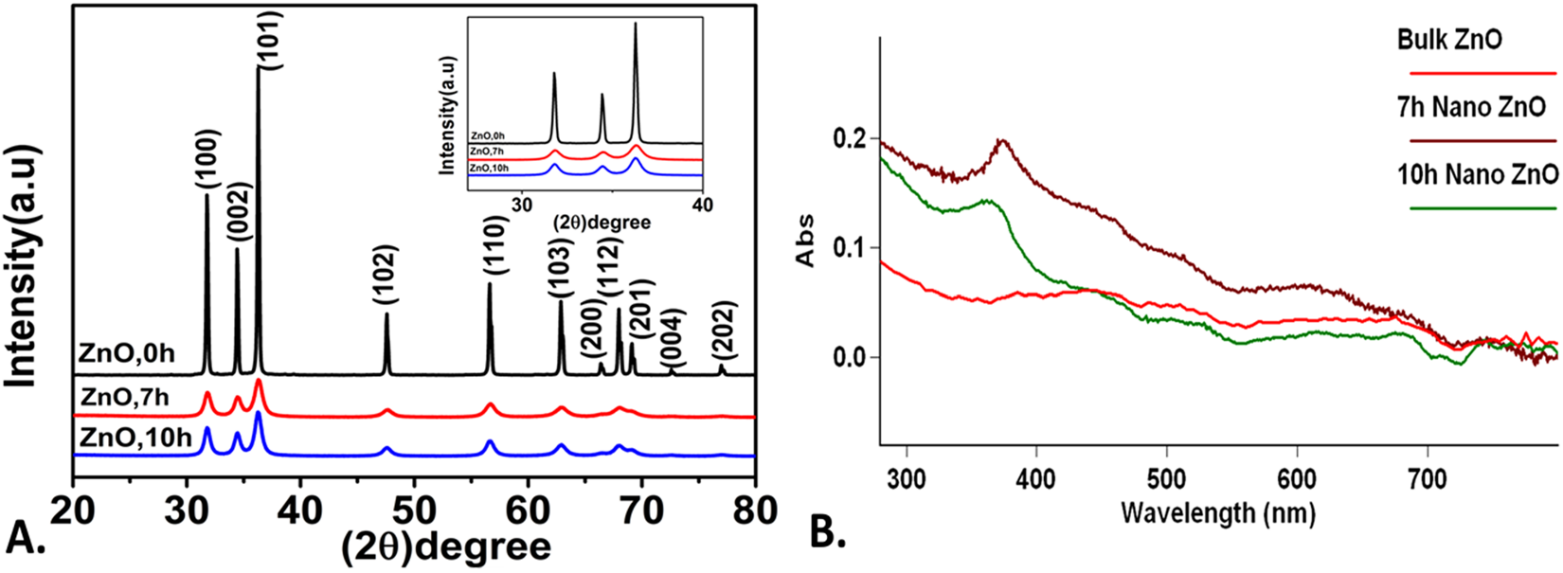
Physiochemical characterization of ZnO nanoparticles milled at different time. (**A**) Structural determination by XRD analysis at 20^0^–80^0^ 2Ѳ (Inset shows diffraction intensity between 30^0^–40^0^ (**B**) Optical characterizations by UV-Vis spectroscopy showing SPR peak shift with increase in milling time.

To analyze the toxicity of industrially synthesized ZnO nanoparticles, HEBM technique was used as prototype of industrial process. ZnO nanoparticles were collected at 7 h and 10 h milling time and characterized for their physiochemical properties by standard techniques. Figure 1 shows size, shape and purity of synthesized ZnO nanoparticles determined by FESEM analysis with EDX. Size of ZnO nanoparticles were found to be decreased with increase in milling time. 7 h milled nanoparticles were 40 nm in size while it was 20 nm in 10 h as compared to the bulk ZnO particle size of 150 nm. EDX analysis confirmed the presence of only ZnO nanoparticles in the synthesized sample. Size and crystallinity of synthesized ZnO nanoparticles were further estimated by XRD analysis. Figure 2A shows the XRD pattern of pure ZnO before ball milling and after milling for 7 h and 10 h. The analysis of the diffraction peak showed the crystal structure of all synthesized powder as hexagonal wurtzite structure (most stable phase of ZnO), belong to the space group of p63mc and matched with JCPDS data card 36–1451^75^. Compare to pure ZnO, the ball milled ZnO showed increased lowering of intensity, broadening of peaks with increase in milling time due to the reduction of crystallite size. The lattice parameter and crystallite size were calculated by Rietveld refinement using maud software and are tabulated in Table 1. The Reitveld method is known as established technique for extracting structural details from the powder diffraction data. This method employs a least squares procedure to compare Bragg intensities and those calculated from a possible structural model^76^. The optical properties of Bulk, 7 h and 10 h ZnO nanoparticles were further determined by UV-Vis spectroscopy. As shown in Fig. 2B, Surface plasmon resonance (SPR) peak of ZnO nanoparticles were found to show blue shift with increase in milling time.

**Table 1:** Structural parameter obtained from XRD analysis of ZnO nanoparticles synthesized by milling at different time.

ZnO with different milling hour	Lattice parameter in (nm)		Tetragonal factor (c/a)	Crystallite size (nm)
	a	c		
Bulk (0 h)	0.32495	0.52061	1.602127	50
7 h	0.32426	0.52022	211.604329	22
10 h	0.32414	0.51967	1.603227	18

**Figure 3:**
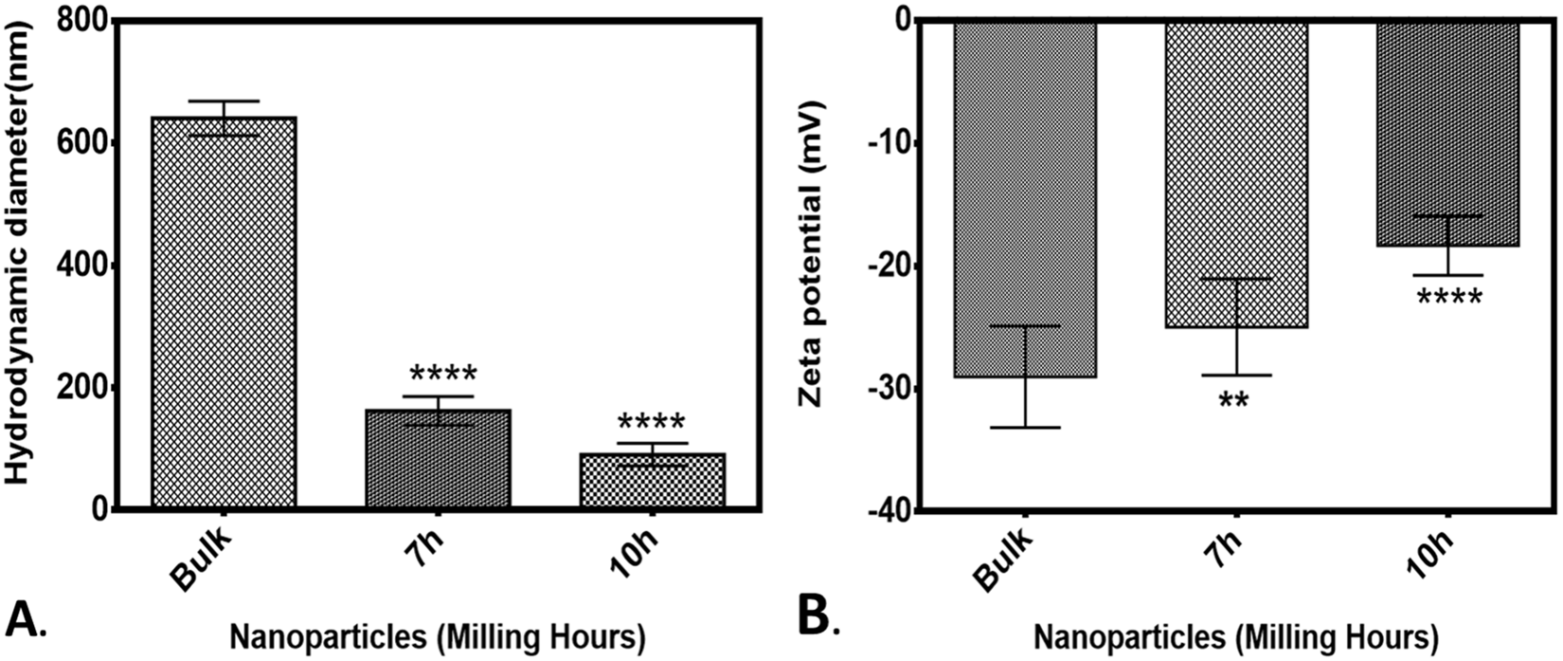
Characterization of ZnO nanoparticles prepared by HEBM at different milling time (Bulk, 7 h and 10 h) using Dynamic light scattering(DLS). (**A**) Hydrodynamic diameter (**B**) Zeta potential. All the parameters were determined by suspending nanoparticles in Holtfreter medium (HF). Measurements were taken in triplicates and data are presented as mean ± SD. Statistical analysis was performed by t-test between Bulk and nano ZnO. Number of (*) present the degree of significance.

It is important to assess the physiochemical properties of nanoparticles in the medium in which their *in vitro* or *in vivo* cytotoxicity is investigated^77^. With this point of view, size and Zeta potential of synthesized ZnO nanoparticles were checked in HF medium which was used in this study to check the effect of nanoparticles in Zebrafish embryos. As shown in Fig. 3A, hydrodynamic diameter of bulk and ZnO nanoparticles were found to be decreased with increase in milling time. The hydrodynamic size of bulk ZnO particles were 640 nm ± 20 nm while it was reduced to 180 nm ± 10 nm and 55 nm ± 5 nm in 7 h and 10 h milled ZnO nanoparticles respectively. Stability of ZnO nanoparticles as determined by their Zeta potential was found to increase with increase in milling time. It was −28 ± 5 mV in bulk ZnO particles which was subsequently increasing to −22 ± 5 mV and −18 ± 4 mV after 7 h and 10 h milling (Fig. 3B). These data confirmed the purity and alteration of physiochemical properties in ZnO nanoparticles with decrease in their size.

### Embryonic toxicity of synthesized ZnO nanoparticles

**Figure 4:**
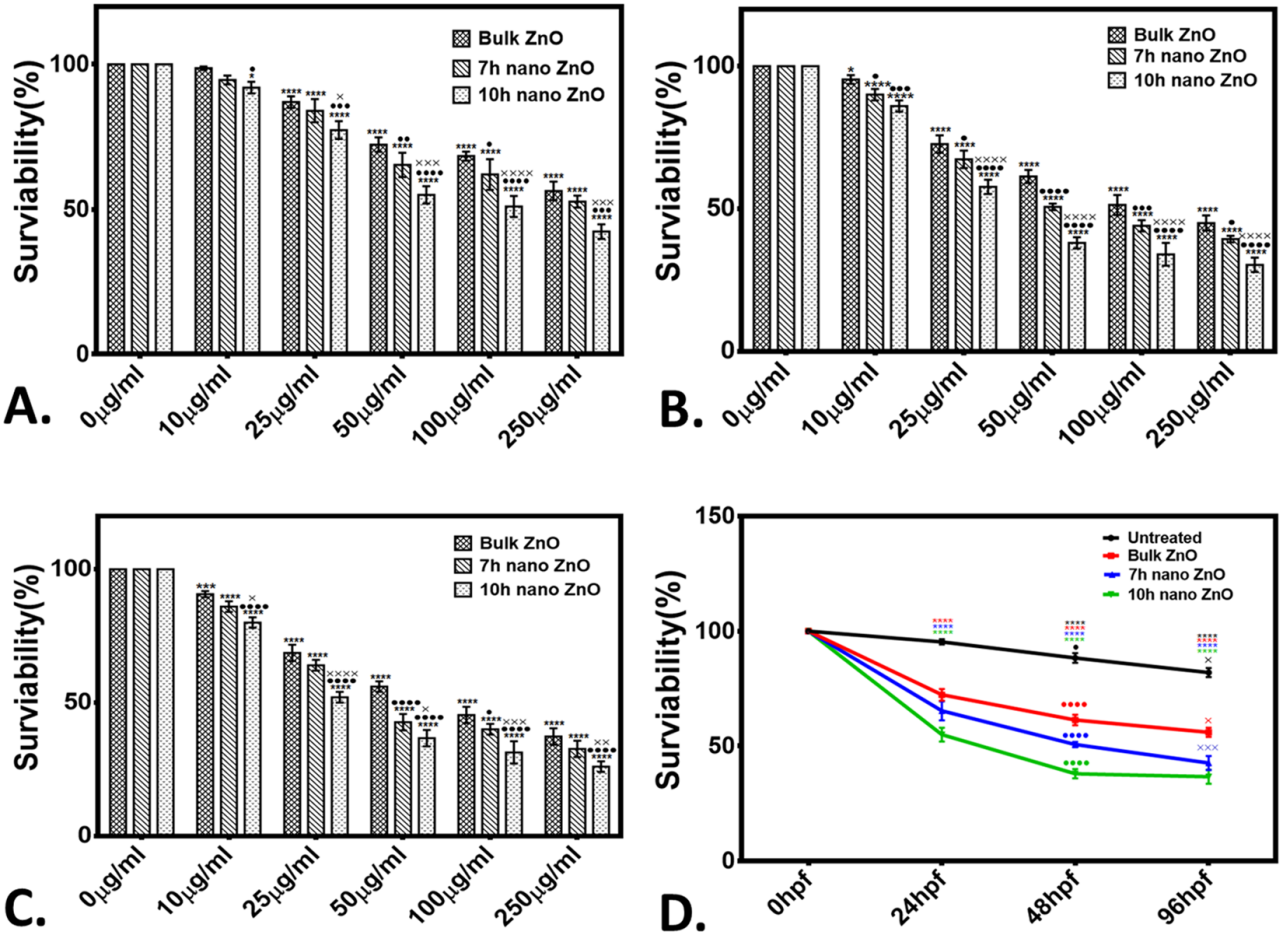
Survivability rate of Zebrafish embryos exposed to different concentration of ZnO nanoparticle (**A**) At 24hpf (**B**) 48hpf (**C**) 96hpf. (**D**) presents comparative analysis at 50 µg/ml ZnO nanoparticles exposure. The values represent mean ± SD of three independent experiments. *P < 0.05, ^•^P < 0.05, ^×^P < 0.05 denotes significant change from untreated control, Bulk and 7 h exposed embryos as obtained from ANOVA analysis. Number of *,^•^,^×^ presents the degree of significance.

**Figure 5:**
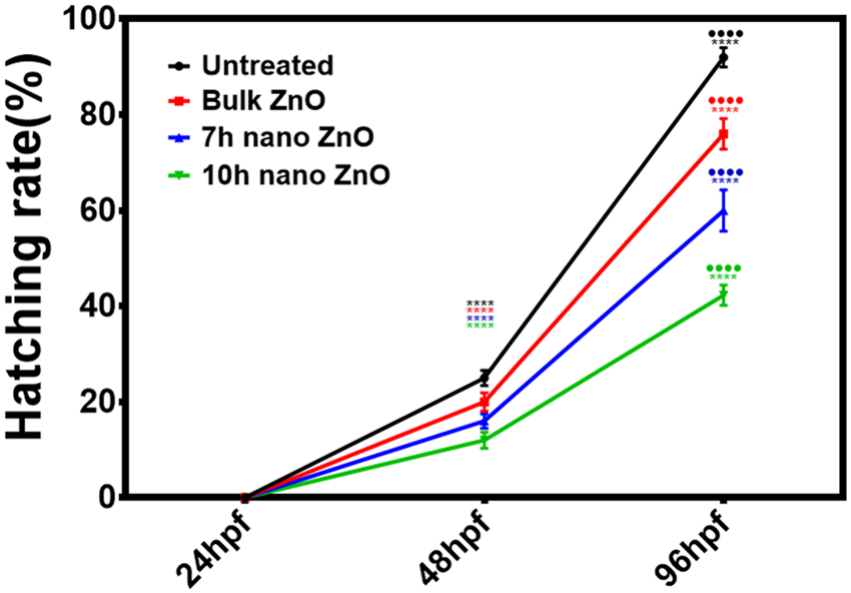
Hatching rate of Zebrafish embryos exposed to 50 µg/ml of Bulk ZnO and ZnO nanoparticles at different hour of post fertilization (hpf). All the measurements were taken in triplicate and the values were presented as mean ± SD of three independent experiments. *P < 0.05, ^•^P < 0.05 denotes significant change from 24hpf and 48hpf embryos respectively as obtained from ANOVA analysis. Number of *,^•^ presents the degree of significance.

**Figure 6:**
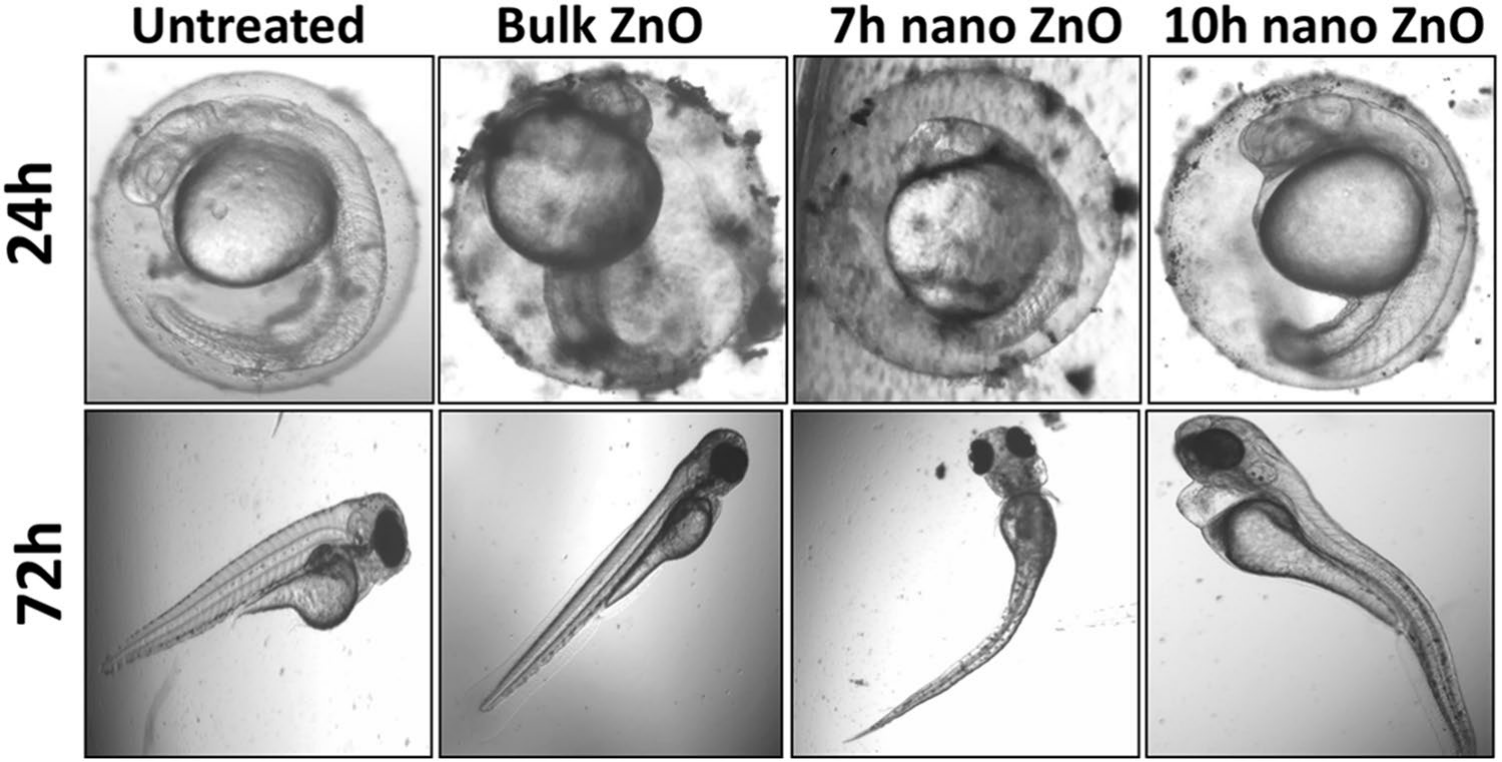
Morphological analysis of Zebrafish embryos exposed to 50 µg/ml of ZnO nanoparticles at different hour post fertilization (hpf). Significant accumulation of Bulk ZnO and nanoparticles were observed at 24hpf. 10 h ZnO nanoparticles exposed embryos showed acute effect with pericardial edema and notochord bending.

The toxicity of industrially synthesized to Zebrafish embryos were studied by investigating toxicological endpoints during 96hpf exposure. Viability of Zebrafish embryos were recorded in presence of different concentration of bulk and 7 h, 10 h ZnO nanoparticles. Survivability rate of embryos exposed to bulk and ZnO nanoparticles were found to be dependent on milling time corresponding to decreasing size and increase charge (Fig. 4). Moreover the Survivability rate was found to be increased with increase in exposure time. As calculated, lowest concentration for 50% survivability (LC_50_) were also found to be decreased with increased milling time. As shown in Fig. 5, hatching rate of embryos was also found to be decreased with increase in milling time of ZnO nanoparticles. It was clearly observed that 10 h milled ZnO nanoparticles inhibited the hatching of embryos up to 50% in a group of 20. The data reflected the interpretations that delay in hatching of Zebrafish embryos in presence of ZnO nanoparticles is a function of charge and size of the ZnO nanoparticles. To observe the morphological deformities and organ malfunctions during exposure of different ZnO nanoparticles, microscopic observation was done and recorded. Figure 6 shows the morphology of 24hpf and 72hpf exposed to 50 µg/ml Bulk ZnO and 7 h, 10 h ZnO nanoparticles. Untreated embryos showed normal development at 24 h and 72 h. Bulk ZnO were found to be accumulated at the chorion of 24 h embryos more intensively in comparison to 7 h and 10 h ZnO nanoparticles. Developments of embryos were also found to be effected as visualized by deformed tail, eye and yolk of bulk and ZnO nanoparticles exposed embryos. Interestingly in 72hpf hatched embryos, Bulk ZnO treated embryo were free from abnormalities and looking similar to untreated one while deformed tail formation with bend body was observed in case of 7 h and 10 h treated embryos. Abnormal body movement was also observed in ZnO nanoparticles treated embryos as a result of developmental deformities.

### Physiological effect of Bulk and ZnO nanoparticles on Zebrafish embryos

**Figure 7:**
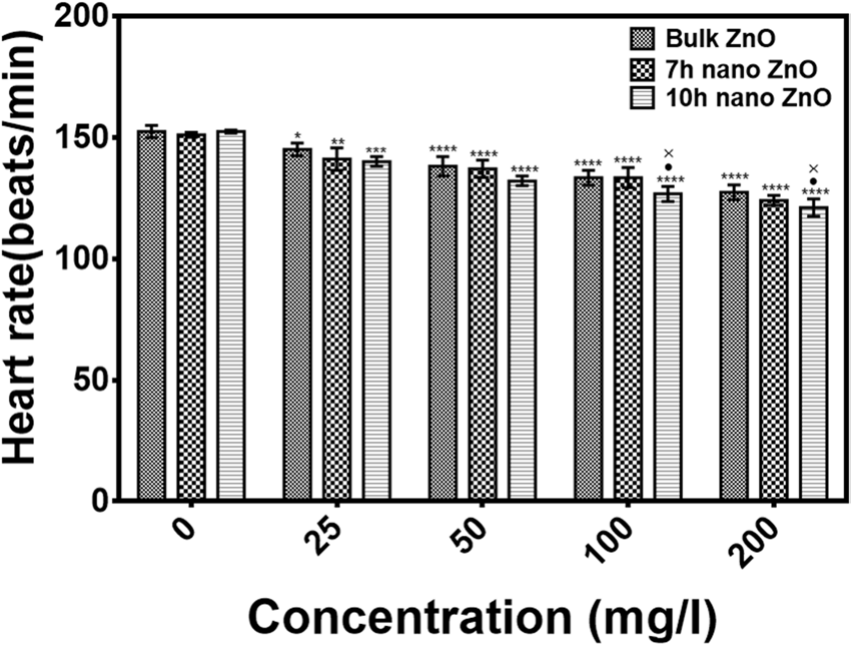
Heartrate of Zebrafish embryos exposed to different concentration of ZnO nanoparticles at 72hpf. All the measurements were taken in triplicate and the values were presented as mean ± SD of three independent experiments. *P < 0.05 shows significant change from untreated control samples. ^•^P < 0.05 and ^**×**^P < 0.05 denotes significant change from Bulk ZnO and 7 h ZnO nanoparticles exposed embryos respectively as obtained from ANOVA analysis. Number of *,^•^,^×^ presents the degree of significance.

Effects of industrially synthesized ZnO nanoparticles on cellular physiology of Zebrafish embryos were investigated first by checking the heart rate of 96hpf Zebrafish embryos treated with Bulk ZnO and 7 h, 10 h ZnO nanoparticles respectively at concentration range of 0–200 µg/ml. As shown in Fig. 7, heart rate of hatched embryos were found to be reduced with increasing concentration of Bulk and ZnO nanoparticles. Interestingly, it was also found to be dependent on milling time of ZnO nanoparticles along with concentration referring to the dependency of physiological toxicity of ZnO nanoparticles on their size and charge.

**Figure 8:**
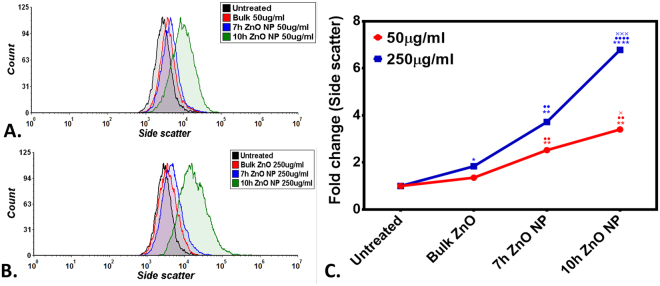
Side scatter analysis done by flow cytometry revealing uptake of Bulk ZnO and ZnO nanoparticles in exposed 72hpf Zebrafish embryos. (**A**) At 50 µg/ml exposure (**B**) At 250 µg/ml exposure. (**C**) Comparative view of fold change in mean side scatter. The values represent mean ± SD of three independent experiments. *P < 0.05, ^•^P < 0.05, ^**×**^P < 0.05 denotes significant change from untreated control, Bulk and 7 h exposed embryos as obtained from ANOVA analysis. Number of *,^•^,^**×**^ presents the degree of significance.

Further to evaluate the intensity of intracellular interaction of ZnO nanoparticles, milling time based uptake kinetics was measured with flow cytometric approach. Figure 8 shows uptake of ZnO nanoparticles in whole body single cell suspension of Zebrafish larvae exposed to 50 µg/ml (Fig. 8A) and 250 µg/ml (Fig. 8B) concentration of Bulk, 7 h and 10 h ZnO nanoparticles by analysis of granularity difference in cells. Granularity of cells as denoted by side scatter of histogram plot were found to be increased with concentration and increase in milling time of ZnO nanoparticles. At 50 µg/ml, fold change in mean side scatter was increasing to 1.3, 2.5 and 3.4 times in Bulk, 7 h and 10 h ZnO nanoparticles exposed larval cells while it was 1.8, 3.7 and 6.8 at 250 µg/ml respectively. The data depicted milling time i.e. size dependent significant change in granularity of cells due to uptake of ZnO nanoparticles.

**Figure 9:**
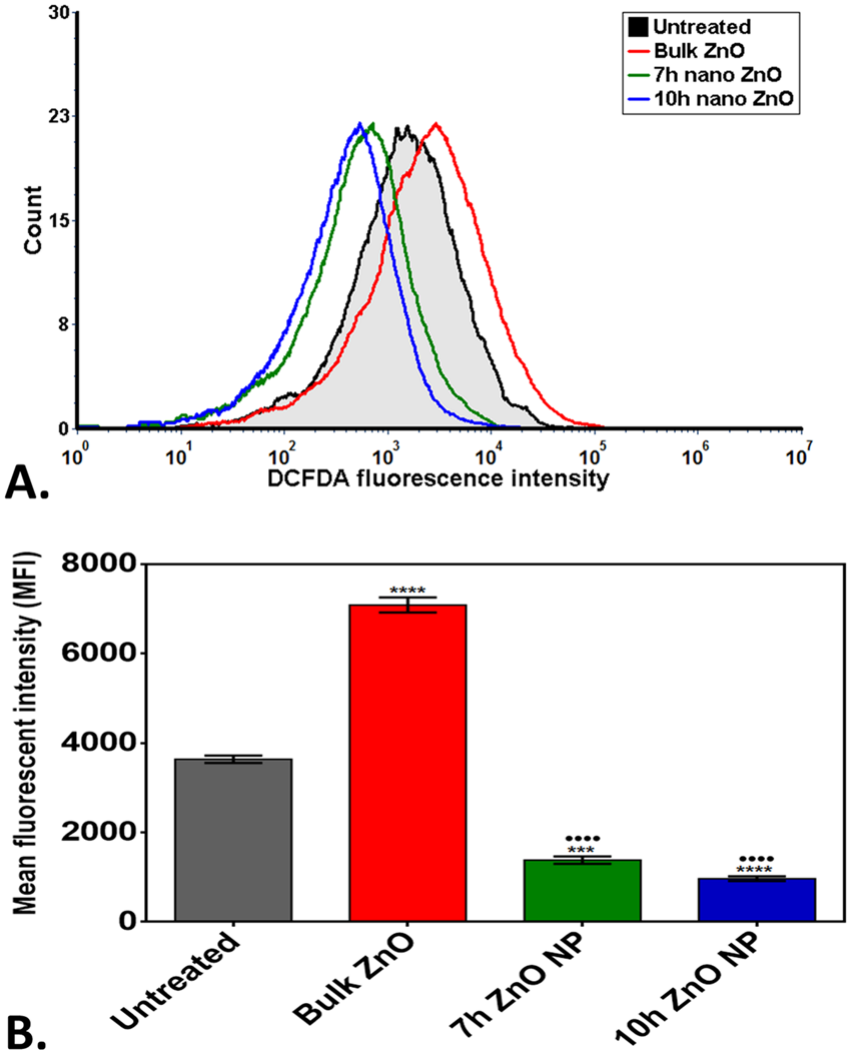
Flow cytometry analysis of ROS alteration in Zebrafish embryos exposed to different concentration of ZnO nanoparticles at 72hpf. Embryos were stained with ROS marker 2,7-dichlorodihydrofluorescein diacetate (H_2_DCFDA). (**A**) Histogram presentation of gated cells (**B**) Mean fluorescence intensity(MFI). All the measurements were taken in triplicate and the values were presented as mean ± SD of three independent experiments. *P < 0.05, ^•^P < 0.05 denotes significant change from Untreated and Bulk ZnO treated embryos respectively. Number of *,^•^ presents the degree of significance.

**Figure 10:**
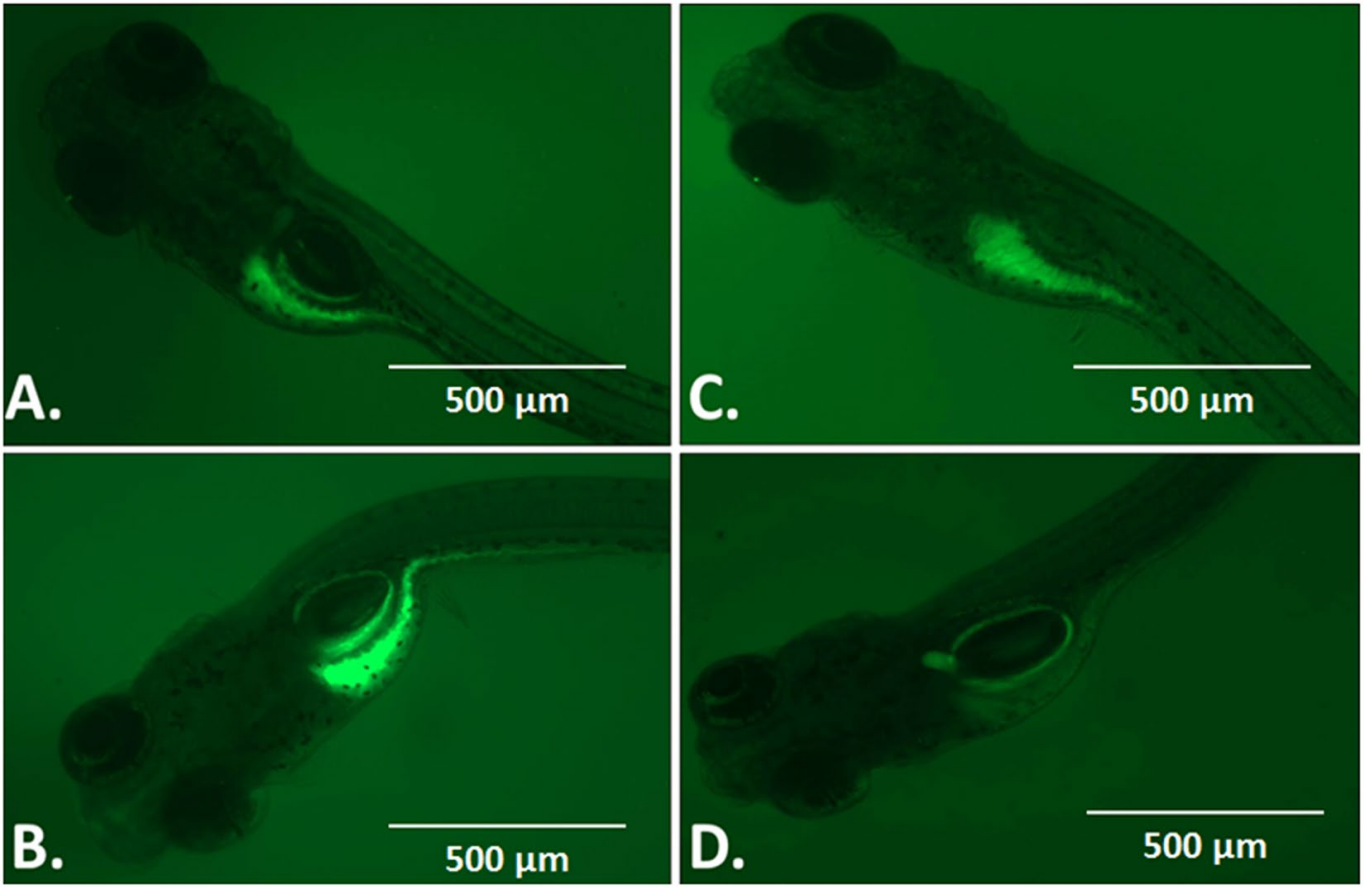
Fluorescent microscopy images of ROS alteration in Zebrafish embryos exposed to ZnO nanoparticles at 72hpf. Embryos were stained with ROS marker 2,7-dichlorodihydrofluorescein diacetate (H_2_DCFDA). (**A**) Untreated Embryo (**B**) Bulk ZnO treated embryo (**C**) 7 h ZnO nanoparticles treated embryo (**D**) 10 h ZnO nanoparticles treated embryo.

**Figure 11:**
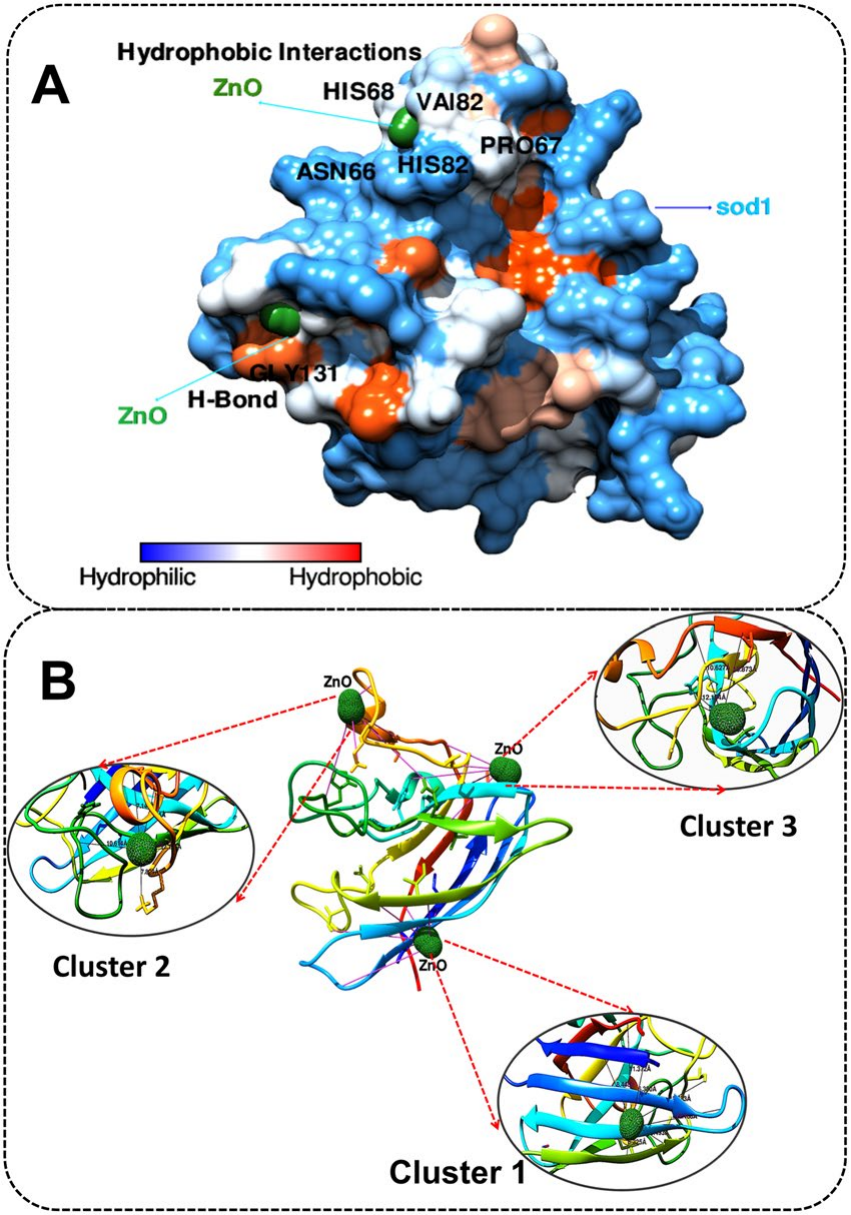
(**A**) Binding confirmation of ZnO nanoparticle with Sod1 protein. (**B**) Molecular interactions of ZnO nanoparticle in different cluster conformations visualized using Chimera.

**Figure 12:**
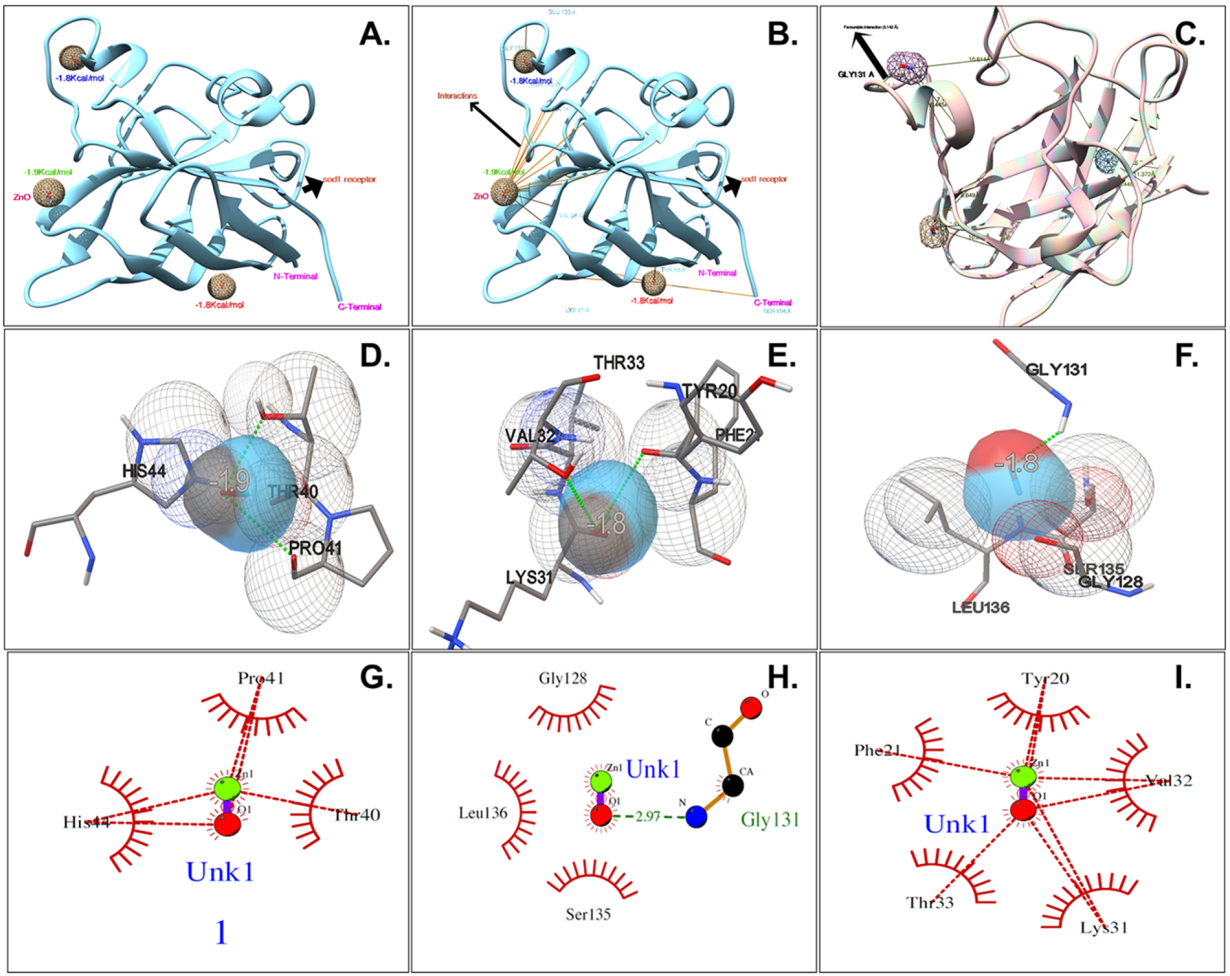
Molecular docking interaction ZnO nanoparticle with Sod1 protein. (**A**) Conformational clusters of ZnO with Sod1. (**B**) Bonding interaction of ZnO clusters (**C**) Favorable binding of clusters. (**D**–**F**) illustrates orientations of three best docking site interaction with minimum binding energy. (**G**,**I**) presents 2D plot of interactions visualized using LigPlot^+^. Unk1 denotes ZnO nanoparticle.

Cellular physiological toxicity of ZnO nanoparticles was further tested by determination of oxidative stress induced by ROS in treated Zebrafish embryos. Figure 9 shows the fluorescence intensity of DCFDA conferring to ROS present in embryonic zebrafish cells treated with 50 µg/ml of Bulk and ZnO nanoparticles. Bulk ZnO were found to induce enhanced generation of ROS in embryonic Zebrafish cells while it was getting quenched in 7 h and 10 h ZnO nanoparticles treated cells respectively. The observation was validated by fluorescence microscopy as shown in Fig. 10. As compared to the untreated Zebrafish larvae higher intensity of DCFDA fluorescence was clearly observed in abdominal region of Bulk ZnO treated Zebrafish larvae which were reducing in 7 h and 10 h ZnO treated fishes in milling time dependent manner. To understand the mechanism of ROS alteration, *in silico* approach was proceeded by molecular docking of ZnO nanoparticles with sod1 protein which is generally recognized as the regulator of oxidative stress proteins in zebrafish^78^. Structure of ZnO was prepared by Autodock by applying Gasteiger charges and merging non-polar hydrogen atoms. While preparation of protein was carried out using standard parameters and by adding Kollman charges^72^ and merging non-polar hydrogen atoms. Figure 11(A) shows conformational probability of ZnO with Sod1 protein. Three probable site of preferable binding was identified as shown in Fig. 11(B) with minimum binding energy of −1.8Kcal/mol and one with −1.9Kcal/mol as shown in Fig. 12(A–C) shows the favorable interaction point of ZnO Np among all three sites which was found at site1 with GLY31 residues. Table 2 represents the molecular docking scores of sod1 with ZnO and also the hydrogen-bonds that are formed with their respective atoms in the amino acids with the help of Chimera^73^. ZnO nanoparticles were interacting with histidine (HIS), threonine (THR) and proline (PRO) residues at site 1 while the interaction was with Glycine (GLY), Leucine (LEU) and Serine (SER) at site 2. At site 3, interaction was found with Valine (VAL), tyrosine (TYR) and Lysine (LYS) residues of Sod1 protein. Orientations of three best docking site interaction with minimum binding energy are presented in Fig. 12(E,D,F). Bonding patterns were predicted in three sites as shown in Fig. 12(G–I). Bonding pattern analysis showed hydrogen bonding with GLY31 residue while other residues were found to interact by hydrophobic bond.

**Table 2:** Molecular docking energies of ZnO nanoparticle interaction with sod1 receptor protein using Autodock Vina showing binding modes and energies in kcal/mol.

Mode	Affinity (kcal/mol)	rmsd (Lower bound)	rmsd (Upper bound)
1	−1.9	0.000	0.000
2	−1.8	22.832	22.832
3	−1.8	20.717	20.717
4	−1.6	14.807	14.807
5	−1.6	10.847	10.847
6	−1.6	22.732	22.732
7	−1.6	16.240	16.240
8	−1.6	23.040	23.040
9	−1.6	10.675	10.675

To determine the consequences of ROS irregularities in embryonic zebrafish cells due to nanoparticles exposure, Apoptosis analysis was done with the help of Acridine orange dye. As shown in Fig. 10, fluorescence of apoptotic cell were seen in untreated zebrafish embryo at abdominal region which were also found in Bulk and ZnO nanoparticle treated embryos. However patches of apoptotic cells were found increased in trunk and tail region of bulk ZnO and 7 h, 10 h ZnO nanoparticles larvae consecutively. The data revealed the milling time i.e. size and charge dependent *in vivo* cellular cytotoxicity of industrially synthesized ZnO nanoparticles in Zebrafish embryos.

## Discussion

This study addressed the issue of *in vivo* cytotoxicity possessed by industrially synthesized ZnO nanoparticles exposed in environment due to their extensive usage in recent scenario. To explore the potential cytotoxicity of industrially prepared ZnO nanoparticles, High energy ball milling technique was used to mimic the mechanical milling process used in industrial synthesis for lab scale synthesis of ZnO nanoparticles. Bulk ZnO particles were milled for 10 h in tungsten carbide container with tungsten carbide balls and toluene medium to provide the inert environment for contamination free synthesis of ZnO nanoparticles. In preparation process, 300 rpm was used to provide ambient rpm and less heat generation to avoid contamination. HEBM technique has been reported as standard green method for synthesis of nanoparticles by many researchers^41^ which also has been utilized by industries now a days for synthesis of different types of nanoparticles^38,79,80^. Synthesis of ZnO nanoparticles from their bulk counterpart by HEBM can be attributed to the high mechanical force generated due to grinding of bulk ZnO particles between WC balls. This approach of synthesizing nanoparticles has been recognized as top down approach in literatures^41^. For cytotoxicity studies, 7 h and 10 h milled ZnO nanoparticles were collected and characterized for their physiochemical properties in HF medium which was used to rear embryonic Zebrafish. FESEM images (Fig. 1) showed significant size decrease in 7 h and 10 h nanoparticles in due to milling of ZnO bulk particles. The purity of synthesized ZnO nanoparticles was confirmed by EDS analysis where peaks of Zn and Oxygen confirmed the presence of ZnO nanoparticles in each case. However a small peak of Silicon was also observed which may be due to the use of silicon chip as substrate for FESEM analysis (Fig. 1b,d,f). Small amount of agglomeration of particles were observed in 7 h and 10 h milled ZnO nanoparticles (Fig. 1). Decrease in size of ZnO nanoparticles from bulk to 7 h and 10 h milling can be attributed to the kinetic energy provided by WC balls to ZnO particles however the tendency of high agglomeration due to energy transfer in smaller molecules can be defied by the fact of brittle nature of ZnO nanoparticles^81,82^. Higher energy produced by movement of balls in container has been reported in literatures for repeated fracturing and welding of metal nanoparticles leading to particle size reduction relying on the time and speed factor^83–85^. Moreover, the ambient environment provided by the presence of toluene also plays important role in prevention of high agglomeration^83,86^. XRD analysis further confirmed decrease in size with maintained hexagonal wurtzite structure of 7 h and 10 h milled ZnO nanoparticles. Clear blue shift in SPR peak while analyzing optical properties with UV-Vis spectroscopy confirmed change in optical properties of ZnO nanoparticles with decrease in size. Appearance of blue shift in SPR peak can be argued to increase in band energy due to electron transfer with decrease in size^87^. Further, Hydrodynamic diameter measurement by using Dynamic light scattering supported the decrease in size of 7 h and 10 h ZnO nanoparticles by milling in HF medium too. However the increased size determined by hydrodynamic diameter measurement in comparison to the FESEM can be reasoned to the attached water and salt molecules with ZnO nanoparticles in the medium^88^. Zeta potential determination revealed increase in charge of ZnO nanoparticles with milling time. Increased positivity of zeta potential with increase in milling time can be attributed to the decreased vacancies in ZnO nanoparticles due to decrease in their size with longer milling time^81^. Investigation of physiochemical properties in synthesized ZnO nanoparticles defined by different experimental data explored the consequent changes with increased milling time. Previous reports have also demonstrated the size dependent changes in physiochemical properties of ZnO nanoparticles synthesized by other techniques^89,90^.

Exploration of potential mode of *in vivo* cytotoxicity of ZnO nanoparticles was done by checking their exposure effect in Zebrafish embryos by different embryo-larvae assays. Results obtained from analysis of survivability in presence of 50 µg/ml of bulk and ZnO nanoparticles revealed milling time i.e. size dependent survivability rate in Zebrafish embryos. 50 µg/ml concentration was chosen considering LC_50_ of the nanoparticles calculated from survivability data of 24hpf, 48hpf and 96hpf exposed embryos. LC_50_ of bulk and 7 h, 10 h ZnO nanoparticles were recorded decreasing with increased milling time. Similar results of increased mortality rate in zebrafish embryos with decrease in size of ZnO nanoparticles have been defined by different toxicologists^59,91^. Hatching study also showed milling time dependent delay in hatching of Zebrafish embryos exposed to ZnO bulk and nanoparticles (Fig. 5). The hatching retardation can be attributed to the size dependent interference of Bulk and 7 h,10 h ZnO nanoparticles with hatching enzymes produced by hatching gland embedded in chorion sac^60^. During normal hatching process hatching gland digest chorion for developmental cycle^92^. Delay in hatching process due to exposure of Bulk and 7 h, 10 h milled ZnO nanoparticles indicated that inhibition of hatching enzyme by ZnO nanoparticles during embryonic development is influenced by the decreasing size and increasing charge. Previous studies have stated the probable process of hatching enzyme inhibition as their interaction with nanoparticles internalizing through the chorion pore canals or the blocking of chorion pores due to their accumulation on the chorion surface leading to oxygen supply shortage which is essential for the embryonic development^93^. Similar results were also mentioned in toxicity studies of Zebrafish embryos with copper nanoparticles^94^ and carbon nanotubes^95^. Delayed hatching activity with increased milling time of synthesized 7 h and 10 h ZnO nanoparticles can be attributed to the enhanced intensity of interaction level due to decrease in size and increase in surface charge of ZnO nanoparticles due to higher milling time. The subsistence of ZnO nanoparticles cytotoxicity to Zebrafish embryos were further investigated to the impacts on physiological process. Acute morphological changes like yolk sac edema and abnormal tail development in 24hpf embryos along with deformed tail and bend body axis in 72hpf hatched embryos were observed after bulk and 7 h, 10 h ZnO nanoparticles. Heartbeat rate were also found to be negatively influenced with increase in milling time of ZnO nanoparticles. Possible cause of these symbolical toxicity features of ZnO nanoparticles exposed embryos could be the size and charge dependent accumulation, internalization and interaction with outer covering, skin and body fluids. Similar description was mentioned by Asharani *et al*.^74^ in case of silver nanoparticles toxicity behavior. Further, to evaluate the effect of changed physiochemical properties (size and zeta potential) in ZnO nanoparticles on cellular physiology of Zebrafish embryos, uptake of nanoparticles were evaluated by measuring granularity change in single cell suspensions. Analysis (Fig. 8) showed milling time and concentration dependent granularity change in embryonic Zebrafish cells depicting the role of altered size and charge in eliciting cellular physiological changes due to ZnO nanoparticles exposure. Elicited physiological metabolic change includes alteration of oxidative stress in form of ROS variation. Flow cytometric analysis of DCFDA fluorescence intensity showed induction of enhanced ROS generation by Bulk ZnO particles with ROS reduction in Zebrafish embryos treated with 7 h and 10 h ZnO nanoparticles (Figs 9 and 10) in comparison to untreated embryos. Previous reports have mentioned enhanced generation of ROS in Zebrafish as well as other live cells due to ZnO nanoparticles exposure^93,96^. This contradictory behavior of HEBM synthesized ZnO nanoparticles can be attributed to the generation of oxygen vacancies due to milling of ZnO particles^81^. Metal Oxide nanoparticles synthesized by Ball milling method has been reported to possess oxygen vacancy as their intrinsic defects by many researchers^97,98^. These oxygen vacancies are the points in the lattice where an electron is missing from the oxygen shell^99^. Such oxygen vacancies can react with the free electrons of free radicals leading to antioxidant property^100^. Previous reports have mentioned quenching of ROS in live models exposed to nanoparticles like cerium oxide^101,102^ and fullerenes^103^ bearing oxygen vacancies. ROS has been reported as the key element of cellular physiological process. It has been regarded as regulator of adaptation to hypoxia in cells^104^ by playing important role in signaling pathways. Moreover they have been recognized as the inducer of autophagy for maintenance of homoeostasis^104–106^. Influential changes in ROS can be attributed to the function of Sod1 protein regulation due to ZnO nanoparticles exposure^78^. To understand the molecular basis of ROS alteration, *in silico* approach was used and molecular docking of ZnO nanoparticles with Sod1 was performed. Docking showed the three favorable interaction modes of ZnO nanoparticles out of 9 modes interpreted with minimum binding energy (Table 2). Autodock predicted hydrophobic interactions with ASN, VAL, HIS and PRO residues while hydrogen bonding was predicted with GLY(Glycine) residue(Figs 11 and 12). Hence it can argued that the altered charge in ZnO nanoparticles due to milling effect influences the configurational changes in Sod1 protein by influencing the interaction with glycine residue and binding energy. This configurational changes downregulates the function of sod1 leading to non-functionality of oxidative stress proteins. It can be argued that the quenching of ROS by bulk and ZnO nanoparticles disturb the normal cellular physiological process by altering the signaling pathways in zebrafish embryos cells which could lead to alteration in other metabolic process promoting in their cytotoxicity.

**Figure 13:**
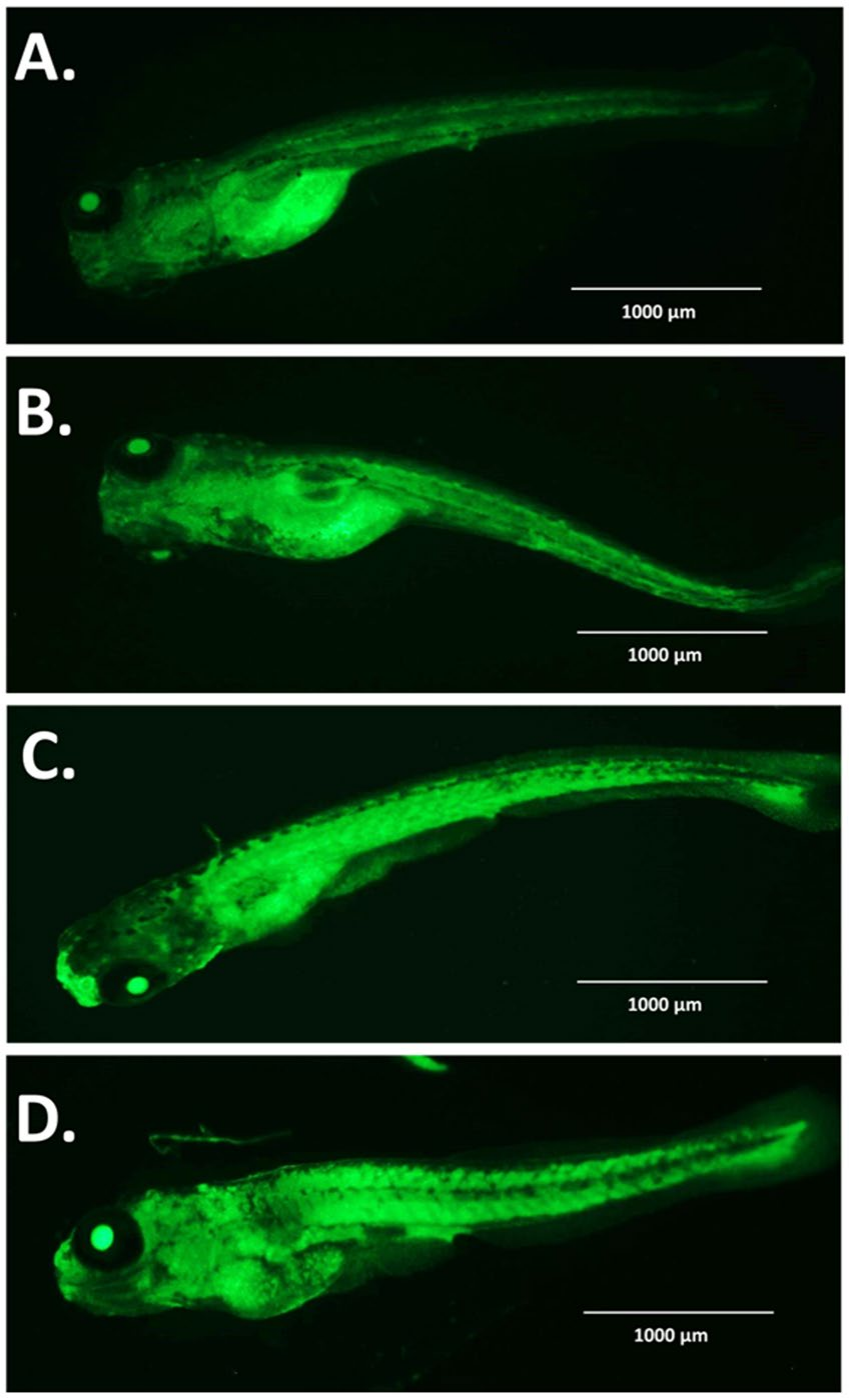
Fluorescent microscopy images of cellular apoptosis in Zebrafish embryos exposed to different ZnO nanoparticles at 72hpf. Hatched Embryos were stained with Apoptosis marker Acridine orange (AO). (**A**) Untreated Embryo (**B**) Bulk ZnO treated embryo (**C**) 7 h ZnO nanoparticles treated embryo (**D**) 10 h ZnO nanoparticles treated embryo.

ZnO nanoparticles have been reported to alter the metabolic process leading to apoptosis and necrosis in live models^107,108^. With reference to the previous reports^109^, it was speculated that HEBM synthesized ZnO nanoparticles would have been eliciting the programmed cell death (apoptosis) in Zebrafish embryos. The expected outcome was tested by apoptosis analysis in hatched embryos by Acridine orange staining. As shown in Fig. 13, Acridine orange staining affirmed the speculation and it was found that the intensity of apoptotic cells in exposed embryos were increasing with increasing milling time of ZnO nanoparticles depicting their dependence on size and charge. With reference to the experimental data and previous reports^93,110^, it can be argued that the exposure of Bulk and ZnO nanoparticles to zebrafish embryos alters their normal physiological and developmental process by interfering with the cellular metabolic pathways. Due to accumulation ZnO nanoparticles on zebrafish embryos cells, hypoxic condition gets created leading to stress conditions. Internalized ZnO nanoparticles quench ROS inside cells making them incapable of regulating the normal signaling pathway for stress regulation. Moreover internalized ZnO nanoparticles interact with apoptosis inducing proteins like p53^111^ influencing their regulation for programmed cell death phenomenon.

This study explained the cytotoxicity of industrially synthesized ZnO nanoparticles to aquatic organism as well as their effect on *in vivo* live model. Observed effects on Zebrafish study can be mimicked to human cells due to their high genetic similarities. Moreover it described a new horizon of the cytotoxicity mechanism. The explanation further needs a detail investigation on proteomics and genomics level for more description.

## Conclusion

**Figure 14:**
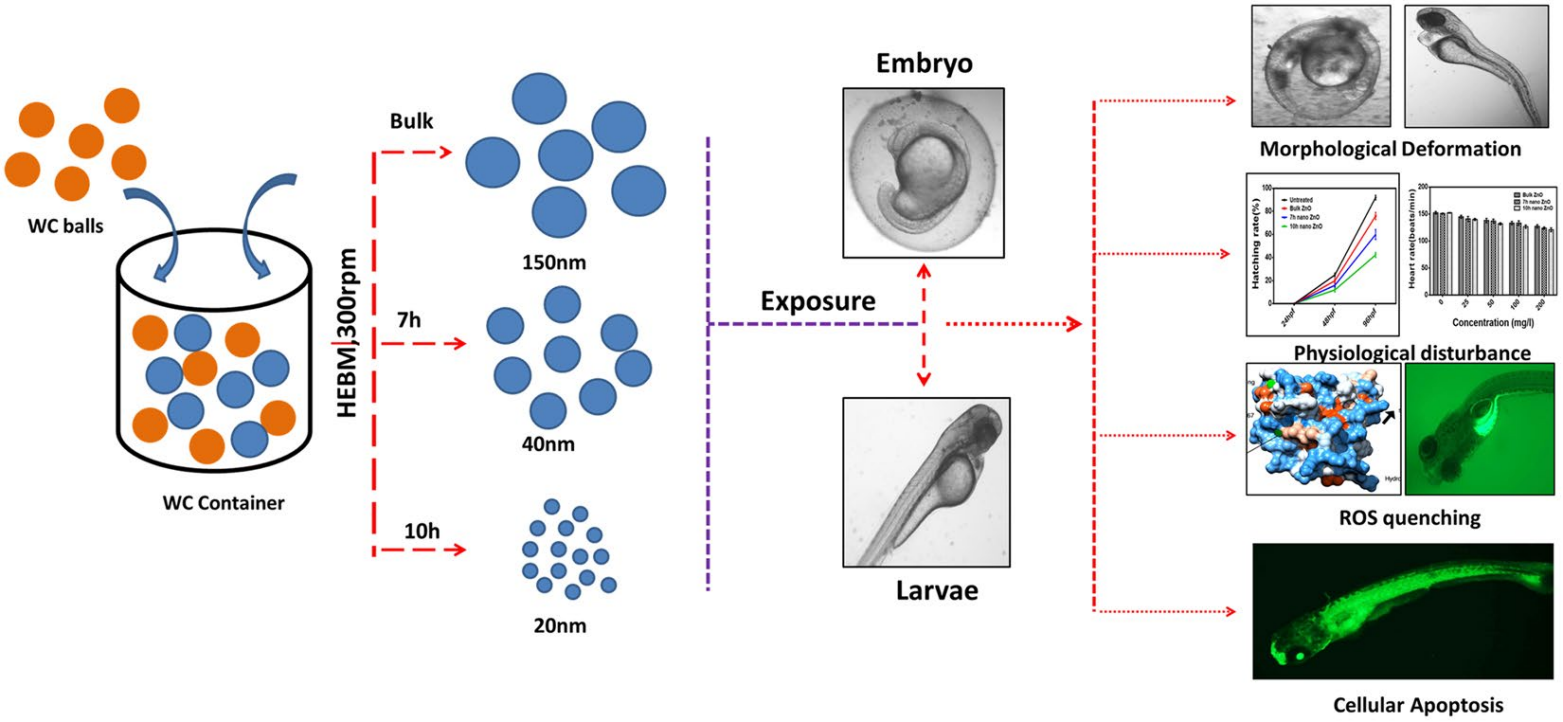
The detailed graphical summary depicting the workflow.

As represented in Fig. 14, this study reveals the *in vivo* cytotoxicity of industrially synthesized ZnO nanoparticles in Zebrafish embryos. Industrial synthesis was mimicked at lab scale by high energy ball milling (HEBM) technique. Synthesized 7 h and 10 h milled ZnO nanoparticles showed significant change in physiochemical and optical properties in comparison to the bulk counterpart. Cytotoxicity evaluation in Zebrafish embryos demonstrated the organ malformation and retarded hatching after 96hpf exposure. Physiological toxicity assessment showed abnormal heart rate on ZnO nanoparticle exposure. Acute ZnO nanoparticles exposure quenched ROS inside cells and induced higher apoptosis. Molecular docking predicted influence of ZnO nanoparticles to Sod1 oxidative stress protein with the help of hydrogen bonding interaction with glycine residues. The result implies the size and charge dependent cytotoxicity of industrially synthesized ZnO nanoparticles. In addition, this study revealed a new aspect of mechanism of cytotoxicity to the aquatic organism and live cells due to ZnO nanoparticles present in industrial products and effluents. Industrially synthesized ZnO nanoparticles were inducing cytotoxicity by accumulating at the surface as well as internalizing inside the cells leading to quenching of ROS interfering with the normal adaptive stress signaling pathway hence increasing the metabolic abnormalities. Moreover internalized ZnO nanoparticles were interfering in normal programmed cell death process influencing the number of apoptotic cells. The study draws attention towards this new aspect of *in vivo* cytotoxicity of industrially synthesized ZnO nanoparticles and demands more investigation to proteomics and genomic level. Currently this aspect of nanotoxicity was hidden regarding the effects of industrial synthesized ZnO nanoparticles. This study will be helpful to understand the mechanism and describe the optimal level of ZnO particles exposure to the daily products and industrial effluents.
